# Triple phase CT in evaluating indeterminate focal liver lesion

**DOI:** 10.6026/973206300222617

**Published:** 2026-04-30

**Authors:** Gaurav Khurana, Rajesh Kumar, Ashish Kumar Shukla, Parinita Sadhanidar, Sachi Mall, Shipra Chaudhary

**Affiliations:** 1 Department of Radiodiagnosis, Santosh Medical College, Santosh Deemed to Be University, Ghaziabad, Delhi NCR, India

**Keywords:** Benign lesions, diagnosis, focal liver lesions (FLLs), multidetector computed tomography (MDCT), triple-phase CT

## Abstract

Indeterminate focal liver lesions (FLLs) detected through ultrasonography or single-phase imaging present a diagnostic challenge due to their varied management depending on whether they are benign or malignant. Therefore, it is of interest to evaluate the diagnostic performance of triple-phase multidetector computed tomography (MDCT) in characterizing these lesions by assessing dynamic contrast enhancement across arterial, portal venous and delayed phases. Results showed that triple-phase CT accurately differentiated benign from malignant lesions, with vascular and enhancement patterns providing key diagnostic insights. Malignant lesions exhibited distinct features, including portal venous hypoenhancement and late-phase washout. Thus, we show the high accuracy of triple-phase CT for managing indeterminate FLLs, supporting better clinical decision-making.

## Background:

Focal liver lesions (FLLs) are frequently encountered in routine clinical practice, either as incidental findings on abdominal ultrasound and CT performed for unrelated complaints or during evaluation of symptoms such as abdominal pain, fever, jaundice, weight loss, or abnormal liver function tests [1]. The clinical challenge lies in the wide differential diagnosis ranging from benign entities such as hemangioma, focal nodular hyperplasia, adenoma, cysts and inflammatory lesions to malignant tumors including hepatocellular carcinoma (HCC), intrahepatic cholangiocarcinoma and metastases [2]. Because management varies from reassurance and follow-up to biopsy, locoregional therapy, systemic therapy, or surgery, timely and accurate characterization is essential to prevent unnecessary interventions for benign lesions and to avoid delays in malignant disease. In real- world settings, a substantial proportion of lesions detected on screening ultrasound or single-phase CT remain "indeterminate," particularly when lesions are small, atypical, or occur in complex clinical backgrounds such as chronic liver disease or known extrahepatic malignancy [3s]. Indeterminate liver lesions represent a specific diagnostic grey zone where imaging does not confidently meet typical criteria for benignity or malignancy. These lesions may remain unresolved because ultrasound is operator-dependent and limited by patient habitus and bowel gas, while non-contrast or single-phase CT may fail to depict the dynamic enhancement characteristics that differentiate lesion types [4]. Even with MRI, certain lesions can remain equivocal due to overlapping appearances, altered hemodynamics, background parenchymal disease, or suboptimal timing and technique. In this context, a robust multiphasic approach is valuable because many hepatic lesions are defined not only by morphology but by vascular behavior over time. The liver receives dual blood supply and focal lesions often show characteristic enhancement patterns depending on arterial supply, portal venous contribution, extracellular space, fibrosis and necrosis. Capturing these evolving patterns is the key principle behind triphasic imaging [5]. Triple-phase computed tomography (triphasic CT) typically includes an unenhanced acquisition followed by arterial phase, portal venous phase and delayed (late) phase imaging. Each phase contributes distinct diagnostic information: arterial phase highlights hypervascularity and tumor neovascularization; portal venous phase provides optimal liver parenchymal enhancement and reveals relative washout or hypoenhancement typical of many malignant lesions; and delayed phase can demonstrate progressive fill-in (classically hemangioma), delayed enhancement of fibrotic tumors and features such as capsule appearance in HCC [6]. Contemporary evidence-based recommendations for characterizing incidentally detected liver lesions recognize multiphasic contrast-enhanced CT as an appropriate second-line modality when initial imaging is inconclusive, with high diagnostic utility in differentiating common benign lesions from malignant disease [7]. CT is a widely available and fast cross-sectional tool, especially relevant in emergency and inpatient settings, with triphasic CT offering enhanced vascular evaluation for focal liver lesions (FLLs). Standardized interpretation frameworks have improved reporting consistency, aiding clinical decision-making [8, 9-1^10^]. For patients at risk of hepatocellular carcinoma (HCC), major imaging features like arterial phase hyperenhancement and washout are critical. In patients with known extrahepatic malignancy, distinguishing metastasis from benign lesions directly impacts treatment [11]. Therefore, it is of interest to determine the role of triphasic CT in accurately characterizing indeterminate FLLs in diverse clinical settings.

## Materials and Methods:

This cross-sectional observational study was conducted in the Department of Radiodiagnosis, Santosh Medical College Hospital (SMCH), Ghaziabad, Uttar Pradesh, India, over duration of 18 months. A total of 64 patients were included. Ethical clearance was obtained from the Medical Research Ethics Committee and written informed consent was taken from all participants prior to enrolment. Patients referred to the Radiodiagnosis department from the Emergency Department of SMCH with suspected liver pathology were assessed for eligibility and recruited according to the predefined criteria. Patients were included if they had clinical suspicion of focal liver lesions, or if a focal liver lesion was detected incidentally on ultrasonography (USG), provided they gave informed consent. Patients were excluded if they had an absolute contraindication to iodinated contrast media or if they were unwilling to participate in the study. All enrolled participants underwent evaluation with triple-phase multidetector computed tomography (MDCT) as part of the study protocol. All examinations were performed on a 192-slice MDCT scanner. Both non-contrast and contrast-enhanced scans were obtained depending on clinical requirements, using Omnipaque™ (iohexol) injection as the iodinated contrast agent. Triple-phase timing included the arterial phase (AP) acquired from approximately 10-25 seconds, the portal venous phase (PVP) beginning around 30 seconds (extending up to ≤120 seconds as applicable) and the late/delayed phase (LP) acquired after 120 seconds and within <5 minutes. Acquisition and reconstruction parameters were standardized: pre-contrast, arterial and portal venous phases were acquired at 5 mm slice thickness with 1-3 mm in-plane resolution and 1 mm reconstruction intervals, while the delayed phase used 5 mm slice thickness, 1-3 mm in-plane resolution and 1-1.5 mm reconstruction intervals. Imaging findings of indeterminate focal liver lesions were assessed, including features suggestive of metastasis and were recorded systematically for analysis. Data were entered in MS Excel 2010 and analyzed using Stata MP-17. Normality of continuous variables was assessed to guide test selection; parametric tests were applied for normally distributed data and non-parametric tests for non-normal data. Descriptive statistics were computed for qualitative and categorical variables and graphical displays were used to facilitate interpretation and presentation of findings.

## Results:

Table 1 (see PDF) summarizes the demographic profile, socioeconomic background and presenting clinical features in the benign (n=28) and malignant (n=36) groups. Males constituted a higher proportion overall (59.4%), with a relatively higher percentage among malignant cases (66.7%) than benign cases (50.0%); however, this difference was not statistically significant (p=0.18). Age distribution was broadly comparable between groups (p=0.29). The largest share of patients belonged to the 40-59-year age group (45.3%), followed by ≥60 years (39.1%) and 18-39 years (15.6%), with malignant lesions appearing slightly more frequent in older patients (≥60 years: 44.4% malignant vs 32.1% benign), though again without statistical significance. Regarding occupation and socioeconomic indicators (Kuppuswamy strata), the pattern was similar in both groups. Skilled/semiskilled work formed the largest category (42.2% overall), followed by clerk/shopkeeper/ farmer (21.9%), unemployed/unskilled (20.3%) and professional/service (15.6%). None of these occupational strata showed significant association with benign versus malignant diagnosis (all p>0.05). Educational status also did not differ significantly (p=0.46): illiterate/primary education was the most common overall (40.6%), followed closely by secondary education (37.5%), while graduate and above accounted for 21.9%. Most patients were married in both groups (overall 90.6%), with no significant difference by diagnosis (p=0.73). Religion distribution was predominantly Hindu (71.9%), followed by Muslim (21.9%), Sikh (4.7%) and others (1.6%) and none differed significantly between benign and malignant categories (all p>0.05). Socioeconomic class was largely concentrated in class III (lower-middle; 39.1%) and classes IV-V (32.8%), again without significant differences between groups (p=0.62). Clinically, right upper quadrant pain was the most frequent symptom (50.0% overall), more common in malignant cases (55.6%) than benign (42.9%), though not statistically significant (p=0.31). Weight loss was the key differentiating symptom: it occurred in 33.3% of malignant cases compared with 10.7% of benign cases, showing a statistically significant association with malignancy (p=0.04). Fever was present in a small minority (15.6% overall) and did not differ between groups (p=0.80). Icterus on examination was more frequent in malignant lesions (25.0%) than benign lesions (7.1%), approaching significance but not meeting the cut-off (p=0.06), suggesting a possible trend toward greater biliary obstruction or advanced disease in malignant cases. Table 2 (see PDF) presents the final diagnostic categorization based on triphasic CT. Among benign lesions, FNH/adenoma/cyst/abscess constituted the majority (57.1% of benign group), while hemangioma accounted for 42.9%. In contrast, malignant lesions were equally split between hepatocellular carcinoma (HCC) (50.0% of malignant group) and metastasis/ cholangiocarcinoma (CCC) (50.0%). The distribution across these categories was highly statistically significant (p=0.001), reflecting that triphasic CT reliably segregated the cohort into distinct benign and malignant diagnostic groups. In the total sample, HCC and metastasis/ CCC each contributed 28.1%, while hemangioma comprised 18.8% and the combined benign non-hemangioma group comprised 25.0%. Table 3 (see PDF) compares lesion size and multiplicity between benign and malignant lesions. Most lesions in both groups fell into the 2.0-4.9 cm range (50% in benign and malignant; p=0.99), indicating this was the most common lesion size category at presentation. Although lesions ≥5 cm were more frequent in malignant disease (38.9%) compared with benign disease (21.4%), the categorical distribution did not reach significance (p=0.12). However, when assessed as a continuous measure, malignant lesions had a significantly larger mean size (4.7 ± 2.3 cm) than benign lesions (3.6 ± 1.8 cm), with p=0.03, suggesting malignant lesions tended to be larger overall even if size categories alone did not fully capture the difference. Multiplicity showed clinically important separation. Solitary lesions were significantly more common in benign cases (78.6%) than malignant cases (52.8%) (p=0.03), supporting the typical pattern of many benign focal lesions presenting as single lesions. Multiple lesions (2-5) were numerically higher in malignancy (33.3% vs 21.4%), though not statistically significant (p=0.27). The "miliary" pattern (>5 lesions) occurred only in the malignant group (13.9%) and was borderline significant (p=0.05), consistent with diffuse metastatic disease patterns. Right lobe predominance was common in both groups (70.3% overall) and did not significantly differ (p=0.36). Table 4 (see PDF) is central to the study objective, demonstrating how enhancement patterns across phases help resolve indeterminate liver lesions. In the arterial phase, the benign group showed a strong association with peripheral nodular enhancement (42.9% benign versus 5.6% malignant; p=0.001), which aligns with the classic arterial enhancement behavior of hemangiomas. Conversely, malignant lesions were strongly associated with heterogeneous hyperenhancement (47.2% malignant vs 10.7% benign; p=0.001), reflecting hypervascular malignant tumors or heterogeneous tumor vascularity. Intense homogeneous enhancement and rim enhancement did not differ significantly (p=0.14 and p=0.17), implying these patterns alone may be less discriminative without considering later phases. In the portal venous phase, benign lesions more commonly showed Isoenhancement (35.7% benign vs 8.3% malignant; p=0.01) and progressive fill-in (35.7% benign vs 5.6% malignant; p=0.002), both of which support benign vascular lesions such as hemangioma or certain benign hypervascular patterns transitioning toward equilibrium. Malignant lesions demonstrated a strong and significant association with hypoenhancement in the portal venous phase (61.1% malignant vs 21.4% benign; p=0.001), consistent with the typical washout tendency of malignant lesions relative to the enhancing liver parenchyma. "Other/mixed/indeterminate" patterns were more frequent in malignant lesions (25.0% vs 7.1%) and were also statistically significant (p=0.04), suggesting that complex or atypical enhancement in PVP is more suggestive of malignancy in an indeterminate lesion cohort. In the late/delayed phase, malignant lesions showed hallmark features: washout (63.9% malignant vs 7.1% benign; p=0.001) and enhancing capsule (30.6% malignant vs 3.6% benign; p=0.006) were both strongly associated with malignancy, which is particularly relevant to diagnosing HCC and other malignant neoplasms. In contrast, benign lesions were significantly associated with delayed progressive fill-in (39.3% benign vs 5.6% malignant; p=0.001), again supporting benign vascular lesions like hemangioma. Capsular contour retraction was seen only in malignant cases (13.9%) and was borderline significant (p=0.05), which can be seen in malignancies such as cholangiocarcinoma or treated lesions and supports malignant etiology when present. Table 5 (see PDF) highlights additional CT features that increase confidence for malignancy beyond enhancement patterns. Satellite nodules were significantly more frequent in malignant lesions (22.2%) than benign lesions (3.6%) (p=0.03), supporting infiltrative or multifocal malignant behavior. Vascular invasion was seen exclusively in malignant cases (19.4%) and in none of the benign cases and this association was statistically significant (p=0.02), making it a highly specific indicator of malignancy in this cohort. Extrahepatic spread was also confined to malignant lesions (16.7% vs 0%; p=0.03), reinforcing its value as an advanced malignant feature. Lymphadenopathy was more common with malignancy (25.0% vs 7.1%) but did not reach statistical significance (p=0.06), suggesting a trend that may become significant with a larger sample size. Table 6 (see PDF) compares the triphasic CT impression (benign vs malignant predicted) with the final diagnosis and demonstrates the overall diagnostic effectiveness of triphasic CT in indeterminate lesions. Triphasic CT correctly identified 26 true benign and 33 true malignant cases. There were 3 false positives (benign predicted but truly malignant) and 2 false negatives (malignant predicted but truly benign). Despite these few discordant cases, the overall diagnostic performance was high: accuracy 92.2%, sensitivity for malignancy 91.7% and specificity for benign disease 92.9%. The reported p-values for sensitivity and specificity (0.002) indicate that the diagnostic classification achieved by triphasic CT was statistically meaningful and unlikely to be due to chance.

## Discussion:

In the present cohort (n=64), baseline demographics showed a mild male predominance overall (59.4%), with males constituting 66.7% of malignant cases versus 50.0% of benign cases, although this difference was not statistically significant (p=0.18). The peak age band was 40-59 years (45.3%), while malignant lesions clustered relatively more in older patients (≥60 years: 44.4% malignant vs 32.1% benign; p=0.29). This pattern is comparable to other Indian triphasic CT series Mittal and Kumbhar (2024) [10] reported a similar male predominance (61.25%) and a tendency toward older age groups, with the commonest decade being 60-69 years (31.25%). In terms of malignant spectrum, our triphasic CT categorization placed half of malignant lesions as hepatocellular carcinoma (HCC) (18/36; 50.0%) and half as metastasis/cholangiocarcinoma (CCC) (18/36; 50.0%). While this "50-50" split reflects the study's indeterminate-lesion referral context, it remains consistent with broader epidemiologic observations that HCC dominates primary liver cancers and is more common in men and older age groups supporting why our malignant arm also leaned male (66.7%) and older (44.4% ≥60 years). McGlynn *et al.* (2021) [11] note that HCC accounts for ~75% of primary liver cancers globally, with incidence typically higher in men than women (commonly around 2-3:1) and rising with age [8]. Clinically, right upper quadrant pain was the most frequent symptom in our cohort (50.0% overall; 55.6% malignant vs 42.9% benign; p=0.31). Weight loss was the key differentiator significantly more frequent in malignant lesions (33.3%) compared with benign lesions (10.7%) (p=0.04), with a near-significant trend for icterus on examination (25.0% malignant vs 7.1% benign; p=0.06). This aligns with symptomatic profiles reported in advanced biliary malignancy cohorts Farhat *et al.* (2008) [12] observed high rates of jaundice (72.7%) and appreciable weight loss (43.6%) and abdominal pain (43.6%), highlighting that constitutional symptoms and cholestasis markers tend to enrich malignant populations and can explain the higher weight-loss signal seen in our malignant group [9]. Our final triphasic CT diagnosis demonstrated a clear categorical segregation: benign lesions comprised hemangioma (12/28; 42.9%) and FNH/adenoma/cyst/abscess (16/28; 57.1%), while malignant lesions comprised HCC (18/36; 50.0%) and metastasis/CCC (18/36; 50.0%) (overall distribution p=0.001). Compared with a broader MDCT characterization study, Jain *et al.* (2019) [13] reported a much higher proportion of benign lesions (72/84; 85.7%) and fewer malignant lesions (12/84; 14.3%), reflecting a more general "all-comers with focal liver lesions" population rather than an indeterminate-lesion enrichment. Their malignant-lesion performance metrics (sensitivity 83.3%, specificity 97.2%, diagnostic accuracy 95.2%) also illustrate that diagnostic yield varies strongly with case-mix, whereas our cohort was intentionally weighted toward more challenging/indeterminate presentations. Lesion burden analysis in our study showed that most lesions were 2.0-4.9 cm (50% in both benign and malignant; p=0.99), yet malignant lesions had a significantly larger mean diameter (4.7 ± 2.3 cm) than benign lesions (3.6 ± 1.8 cm) (p=0.03). Multiplicity also separated groups: solitary lesions were commoner in benign disease (78.6%) than malignant disease (52.8%) (p=0.03) and a miliary pattern (>5 lesions) occurred only in malignant lesions (13.9%; p=0.05). This is clinically coherent with metastatic biology and surgical-planning literature Vialle *et al.* (2016) [14] showed that multiphase MDCT detected 127/158 colorectal liver metastases (sensitivity 80.4%) and identified additional metastases in 20% of patients that altered surgical strategy, underscoring why multiplicity detection is central and why extensive lesion burden tends to track with malignant etiologies. Arterial-phase (AP) behavior in our cohort strongly supported benign-malignant discrimination when interpreted with later phases. Peripheral nodular enhancement was highly associated with benign lesions (42.9% benign vs 5.6% malignant; p=0.001), consistent with hemangioma physiology, while heterogeneous hyperenhancement was strongly linked to malignancy (47.2% malignant vs 10.7% benign; p=0.001), reflecting tumoral neovascularity and necrosis/heterogeneity. These observations mirror the classic multiphasic "pattern-based" approach: van Leeuwen *et al.* (1996) [15] described 11 enhancement patterns in triphasic spiral CT across 94 patients and 375 lesions, with several patterns being consistently benign and others consistently malignant, supporting the concept that multiphasic enhancement pattern recognition is the cornerstone of CT characterization-exactly the diagnostic principle operationalized in our (Table 4 - see PDF) results [12]. Portal venous phase (PVP) findings further strengthened classification in our study: malignant lesions were predominantly hypoenhancing (61.1% malignant vs 21.4% benign; p=0.001), whereas benign lesions more often showed isoenhancement (35.7% benign vs 8.3% malignant; p=0.01) and progressive fill-in (35.7% benign vs 5.6% malignant; p=0.002). Late/delayed phase (LP) findings provided the most decisive malignant signatures in our cohort: washout (63.9% malignant vs 7.1% benign; p=0.001) and enhancing capsule (30.6% malignant vs 3.6% benign; p=0.006) were strongly associated with malignancy, while delayed progressive fill-in favored benignity (39.3% benign vs 5.6% malignant; p=0.001). Ancillary signs further increased specificity-vascular invasion (19.4% malignant vs 0% benign; p=0.02) and extrahepatic spread (16.7% malignant vs 0% benign; p=0.03) were exclusive to malignant disease in our dataset. These findings are concordant with standardized diagnostic frameworks Alhasan *et al.* (2019) [16] quantified LI-RADS CT major feature performance for HCC, showing high sensitivity for arterial hyperenhancement (86.1%) and washout (81.6%), with lower sensitivity but high specificity for capsule (sensitivity 20.7%, specificity 89.9%). The relatively high capsule prevalence in our malignant cohort (30.6%) is therefore plausible in an enriched malignant case-mix and highlights why combining washout + capsule + ancillary invasive signs yields stronger confidence than any single sign alone. Overall, triphasic CT performance in our indeterminate-lesion cohort was high: 26 true benign and 33 true malignant classifications with only 3 false positives and 2 false negatives, yielding accuracy 92.2%, sensitivity for malignancy 91.7% and specificity 92.9%. This is closely comparable to established diagnostic performance in prior triphasic CT work; Kaushal *et al.* (2016) [17] reported triphasic CT sensitivity 91.3% and specificity 97.8% for hepatic lesion evaluation (with strong agreement statistics), supporting that-when multiphasic protocols are optimized CT can consistently provide robust benign-malignant separation. Our slightly lower specificity relative to that report is reasonably attributable to the deliberate inclusion of indeterminate lesions and mixed malignant pathologies, which typically increases borderline and overlap patterns.

## Conclusion:

Triple-phase MDCT demonstrated high accuracy, sensitivity and specificity in characterizing indeterminate focal liver lesions, particularly distinguishing malignant from benign lesions. Malignant lesions were associated with specific imaging features like portal venous hypoenhancement and late-phase washout, while benign lesions exhibited peripheral nodular enhancement. Overall, triphasic CT effectively reduced diagnostic uncertainty, aiding in appropriate management decisions.
